# Deep Sequencing of Plasma Exosomal microRNA Level in Psoriasis Vulgaris Patients

**DOI:** 10.3389/fmed.2022.895564

**Published:** 2022-05-19

**Authors:** Xiu-Min Chen, Dan-Ni Yao, Mao-Jie Wang, Xiao-Dong Wu, Jing-Wen Deng, Hao Deng, Run-Yue Huang, Chuan-Jian Lu

**Affiliations:** ^1^State Key Laboratory of Dampness Syndrome of Chinese Medicine, The Second Affiliated Hospital of Guangzhou University of Chinese Medicine (Guangdong Provincial Hospital of Chinese Medicine), Guangzhou, China; ^2^The Second Affiliated Hospital, Guangzhou University of Chinese Medicine (Guangdong Provincial Hospital of Chinese Medicine), Guangzhou, China; ^3^Guangdong Provincial Key Laboratory of Chinese Medicine for Prevention and Treatment of Refractory Chronic Diseases, Guangzhou, China; ^4^Guangdong-Hong Kong-Macau Joint Lab on Chinese Medicine and Immune Disease Research, Guangzhou University of Chinese Medicine, Guangzhou, China

**Keywords:** psoriasis vulgaris, plasma, exosome, miRNA, inflammatory response, metabolism

## Abstract

Psoriasis is a chronic skin disease affecting 1% to 3% of the world population. Psoriasis vulgaris (PV) is the most common form of psoriasis. PV patients suffer from inflamed, pruritic and painful lesions for years (even a lifetime). However, conventional drugs for PV are costly. Considering the need for long-term treatment of PV, it is urgent to discover novel biomarkers and therapeutic targets. Plasma exosomal miRNAs have been identified as the reliable biomarkers and therapy targets of human diseases. Here, we described the levels of plasma exosomal miRNAs in PV patients and analyzed the functional features of differently expressed miRNAs and their potential target genes for the first time. We identified 1,182 miRNAs including 336 novel miRNAs and 246 differently expressed miRNAs in plasma exosomes of healthy people and PV patients. Furthermore, the functional analysis found differently expressed miRNA-regulated target genes enriched for specific GO terms including primary metabolic process, cellular metabolic process, metabolic process, organic substance metabolic process, and Kyoto Encyclopedia of Genes and Genomes (KEGG) pathway containing cellular processes, human diseases, metabolic pathways, metabolism and organismal systems. In addition, we found that some predicted target genes of differentially expressed miRNAs, such as CREB1, RUNX2, EGFR, are both involved in inflammatory response and metabolism. In summary, our study identifies many candidate miRNAs involved in PV, which could provide potential biomarkers for diagnosis of PV and targets for clinical therapies against PV.

## Introduction

Psoriasis is a chronic, inflammatory, systemic skin disease (1, 2). 1% to 3% of the world population suffers from psoriasis (3). PV is the most common form of psoriasis occurred in 80%–90% psoriasis patients who manifest erythematous papules covered with pearly scales on extensor surface of extremities, scalp and sacral region (1, 4). Due to its prevalence, diversity and duration, scientists and medical workers pay more and more attention to discovering novel biomarkers and therapeutic targets for psoriasis vulgaris.

As membrane-bound nanovesicles of 30–100 nm in diameter, exosomes are secreted by most cell types and exit in almost all bodily fluids (5–7). Various components including lipid, protein, mRNAs, microRNAs (miRNAs), long non-coding RNA (lncRNA) have been identified in exosomes (5, 6, 8). Numerous studies have indicated that recipient cells can be regulated by the above exosomal RNAs through the uptake of circulating exosomes (9, 10). Of these, circulating exosomal miRNAs have been identified as the reliable biomarkers and therapy targets of human diseases, such as cancers, respiratory illness, diabetic nephropathy and autoimmune diseases (11–14). For example, the serum exosomal miR-24-3p level in nasopharyngeal carcinoma has been revealed to correlate with worse disease-free survival of patients (15). Furthermore, plasma exosomal miRNA miR-126 have potential to predict acute respiratory distress syndrome (16). A subset of serum exosomal miRNAs (miR-4449, miR-642a-3p, miR-1255b-5p, let-7c-5p, miR-1246, let-7i-3p, miR-5010-5p, miR-150-3p) associate with diabetic nephropathy (17). More importantly, a recent study has demonstrated that extrinsic microRNA *let-7i* transferred by plasma exosomes might have an active role in triggering autoimmune diseases (18).

Considering that circulating exosomal microRNAs modulate immune response (11), plasma exosomal microRNAs might have the potential to predict immune disorders including psoriasis vulgaris. In this study, the high-throughput RNA sequencing was employed to identify differentially expressed plasma exosomal miRNAs in patients with psoriasis vulgaris, and results were validated by quantitative real-time polymerase chain reaction (qRT-PCR). Subsequently, the enrichment analysis of the GO term and Kyoto Encyclopedia of Genes and Genomes (KEGG) for target genes of differently expressed miRNA were conducted to provide insights exploring reliable candidates for the diagnosis and treatment of psoriasis vulgaris.

## Materials and Methods

### Ethics Statement

All experimental procedures of the present study were approved by the Institutional Review Board of Guangdong Provincial Hospital of Chinese Medicine and conducted in accordance with the Declaration of Helsinki (#B2014-029-01). Written informed consent was obtained from all recruited participants.

### Patients

The clinical characteristics of 15 healthy people and 15 PV patients recruited for this study were shown in Table 1. All selected PV patients fulfilled the Clinical Guidelines of Psoriasis 2008 formulated by the Chinese Medical Association (19). The inclusion criteria were: (1) patients meeting diagnosis standards of PV; (2) patients corresponding the progressive stage of PV; (3) patients diagnosed by two clinicians regarding relevance to PV. Besides, patients combined with tumor, serious cardiovascular, liver and kidney comorbidities, hematopoietic system disease, high fever, tuberculosis, acute suppurative and other infectious diseases were excluded. In addition, women in pregnancy and lactation were also excluded. Fasting venous blood was drawn and centrifuged, then the separated plasma was stored at −80° until detection.

**TABLE 1:** Demographic characteristics of psoriasis vulgaris (PV) patients and healthy control.

Variable	Healthy control (*n* = 15)	PV (*n* = 15)
Age, years^#^	49.33 (8.15)	53.73 (14.88)
**Age, group, n**		
≤ 25	0	1 (6.67%)
26–40	2 (13.33%)	2 (13.33%)
41–55	10 (66.67%)	4 (26.67%)
≥ 56	3 (20.00%)	8 (53.33%)
**Sex, n**		
Male	9 (60.00%)	11 (73.33%)
Female	6 (40.00%)	4 (26.67%)
**PASI**		
0	15 (100.00%)	0 (100.00%)
1	0	0
2	0	5 (33.30%)
3	0	3 (20.00%)
4	0	4 (26.67%)
5	0	2 (13.33%)
6	0	1 (6.67%)

*^#^Age data are presented as the mean (SD). PV: psoriasis vulgaris; HC: healthy controls.*

### Exosome Isolation

Exosomes were isolated from 500 μl plasma samples according to the manufacturer’s protocols using Exo Quick Exosome Precipitation Solution Kit (20), and identified by scanning electron microscopy (SEM) (FEI XL30, The Netherlands) with low-voltage (1 KeV) and magnification of 20,000, NanoSight and Western blot analysis in our previous study (20), which shared exosomes used in the present study.

### Small RNA Library Construction, Sequencing, and miRNA Identification

After the extraction of total RNA from plasma exosome by TRIzol (Thermo Fisher Scientific, Waltham, MA, United States), the RNAs ranged from 18 to 30 bp were enriched. Then adapters were ligated to RNAs followed by the reverse transcription of adapter-ligated RNAs, and the 140–160 bp size products were collected for the construction of cDNA library and sequencing by Illumina HiSeq™ 4000.

Subsequently, raw reads were analyzed by in-house Perl scripts to collect clean tags. After discarding dirty reads with over 10% poly-N sequences or whose Phred scores were < 5%, all clean tags were aligned with miRNAs using GeneBank database and Rfam database (v11.0). Besides, all clean tags were also aligned with human reference genome (Grch37) utilizing TopHat v2.0.9 (21). Next, clean tags were blasted in miRBase database (v21) to screen known miRNAs. Moreover, all unannotated tags were predicted using Mireap_v0.2 software based on their genome positions and hairpin structures to identify novel miRNA candidates.

### miRNA Levels

The levels of total miRNAs were calculated and normalized to transcripts per million (TPM) using the following formula: TPM = Actual miRNA counts/Total counts of clean tags × 10^6^. Besides, levels of miRNAs in different groups were displayed by the heatmaps to cluster miRNAs with similar level patterns.

### Analysis of miRNA Differential Levels

The formula used to determine miRNA differential levels across groups was shown as follows:


$$p\,\left(x|y\right)={\left(\frac{N_{2}}{N_{1}}\right)}^{{}^{y}}\,{\frac{\left(x+y\right)!}{x!\,y!\,{\left(1+\frac{N_{2}}{N_{1}}\right)}^{\left(x+y+1\right)}}}_{D\left(y\geq y_{\max}|x\right)=\sum_{y\geq y_{\max}}^{\infty }p\left(y|x\right)}^{C\left(y\leq y_{\min}|x\right)=\sum_{y=0}^{y\leq y_{\min}}p\left(y|x\right)}$$

Besides, miRNAs with a fold change (FC) ≥ 2 and *P* value < 0.05 in a comparison were considered as significant differently expressed miRNAs.

### Prediction of Target Genes of Differently Expressed miRNAs

The candidate target genes of differently expressed miRNAs of miRNAs were predicted by RNAhybrid (v2.1.2), Miranda (v3.3a) and TargetScan (v7.0) software based on sequences. Besides, the miRNA-target gene network was established using Cytoscape software (v3.6.0).

### Gene Ontology and Kyoto Encyclopedia of Genes and Genomes Pathway Enrichment Analysis for Target Genes

All target genes of different expressed miRNAs were mapped to GO terms based on Gene Ontology database. Besides, significantly enriched GO terms were identified by hypergeometric test. KEGG is an important public pathway-related database. Therefore, KEGG was used for analyzing pathway enrichment to determine significantly enriched pathways for target genes of different expressed miRNAs.

### Validation of Small RNA Sequencing Data by Quantitative Real-Time Polymerase Chain Reaction

Quantitative real-time polymerase chain reaction (qRT-PCR) assays were performed to confirm the reliability of the small RNA-seq data according to previous studies (22, 23). Small RNA was reversed transcripted by the miRcute miRNA First-Strand cDNA Synthesis Kit (Tiangen, Beijing, China). Besides, miRNA levels were normalized to the level of U6 according to the ΔΔCT method.

### Statistical Analysis

Statistical differences of data in this study were analyzing by the unpaired Student’s *t*-test between two groups using SPSS software (v20.0, SPSS Inc., Chicago, United States). Besides, *P* < 0.05 indicated statistically significant.

## Results

### Analysis of Small RNA Sequencing

Thirty small RNA libraries, including 15 PV samples (PV1-15) and 15 samples (HC1-15), were constructed and sequenced to reveal miRNA profiles. After the filter of low-quality reads, approximately 13 million clean tags were obtained from PV groups while the number of clean tags obtained from control groups was about 11 million. The percentage of clean reads in each group was approximately 88%. Then, clean reads were mapped to the human reference genome (Grch37) by TopHat. Results showed that more than 90% of clean reads were mapped.

### Identification of miRNA

**FIGURE 1:**
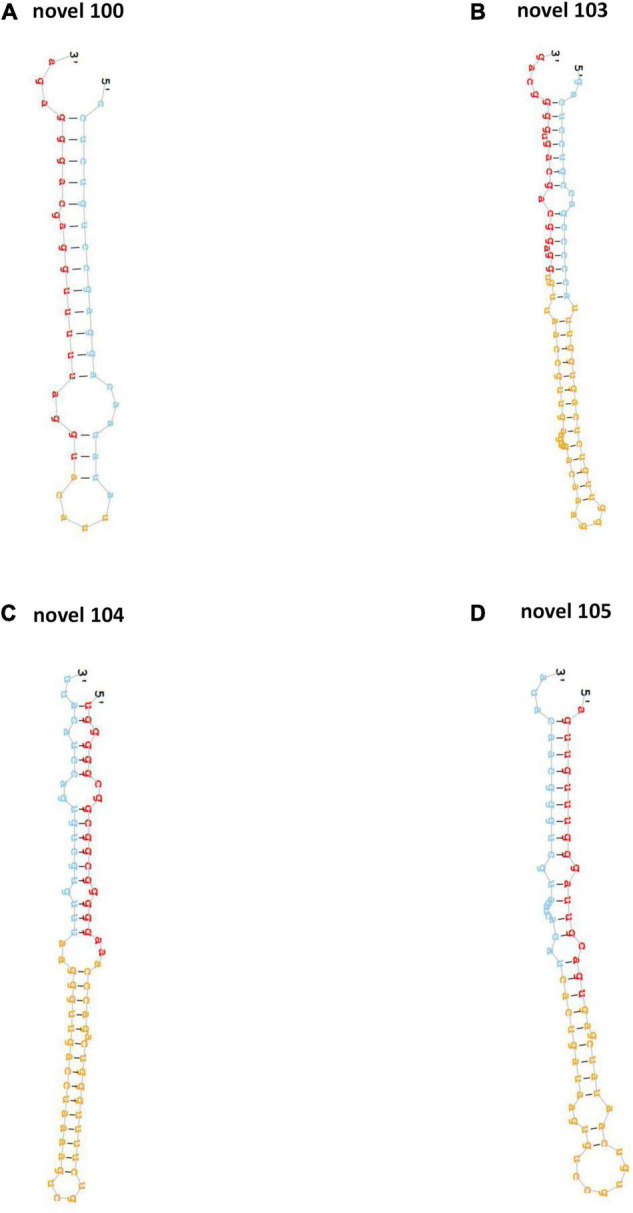
The hairpin structures of four novel precursor miRNAs. The secondary structures of four novel precursor miRNA identified in this study, including novel-100 **(A)**, novel-103 **(B)**, novel-104 **(C)** and novel-105 **(D)**.

After the alignment with GenBank and Rfam (11.0), rRNA, scRNA, snoRNA, snRNA, tRNA were removed from clean tags. Results of the mapping to human reference genome revealed that 751 and 846 known miRNAs were found in clean tags of control and PV groups, respectively (Supplementary Table 1). Moreover, 257 and 336 novel miRNAs were uncovered from clean tags of in clean tags of control and PV groups, respectively (Supplementary Table 2). The hairpin structures of four novel precursor miRNAs (novel 100, novel 103, novel 104, novel 105) were displayed in Figures 1A–D.

### miRNA Differential Levels

**FIGURE 2:**
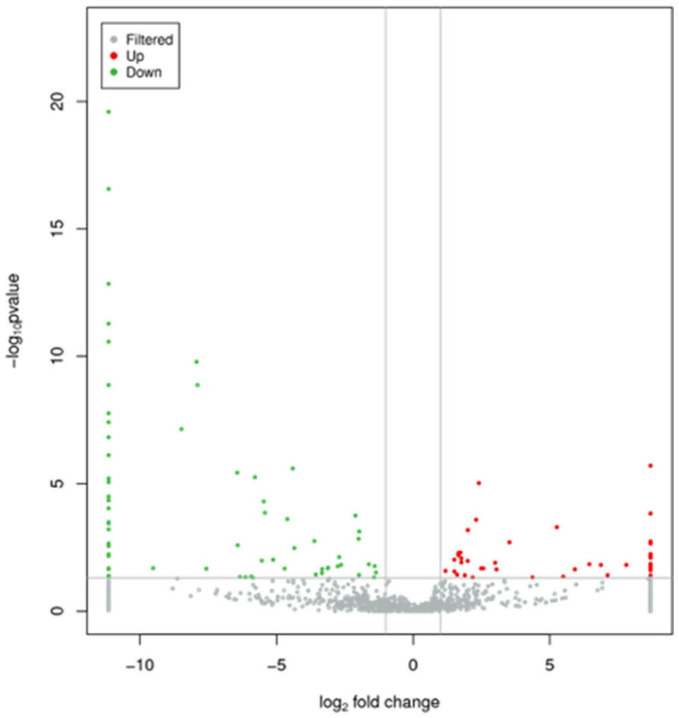
Characteristics of miRNA levels between control group and PV group. All miRNA levels are shown, and miRNAs with differentially levels are shown in red (up-regulated) or green (down-regulated).

**FIGURE 3:**
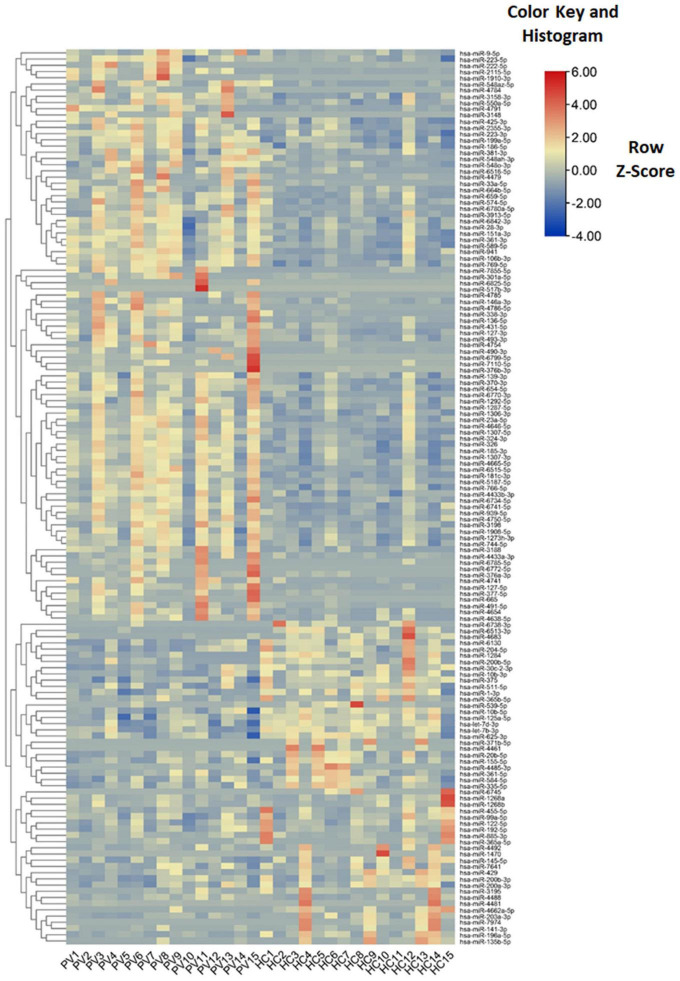
Characteristics of miRNA levels between different groups. Heat map showing the levels of miRNAs (*P* < 0.05) in different groups. Colors from blue to red stand for z-score got through the dimensionality reduction of FPKM value and reveal decreasing miRNA levels in each group.

**FIGURE 4:**
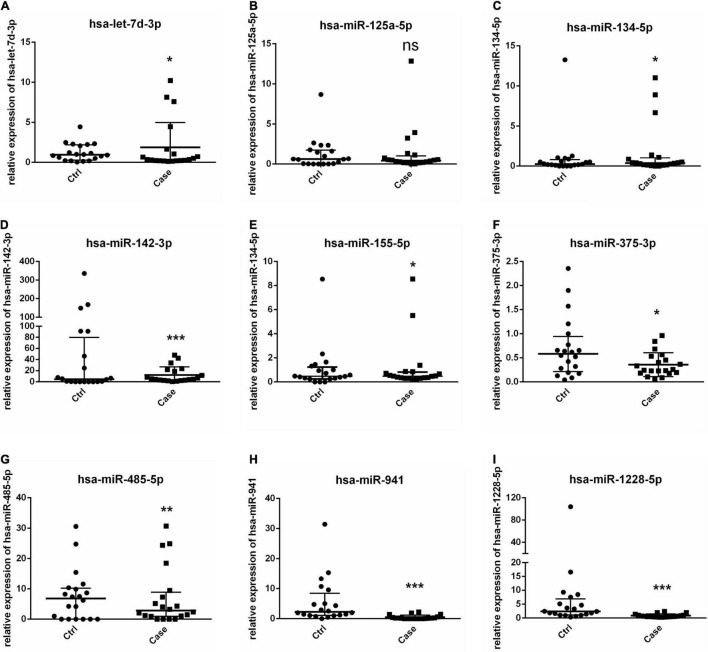
Validation of miRNAs by RT-PCR. **(A–I)** Levels of 9 selected miRNAs are determined by RT-PCR. * *P* < 0.05, ** *P* < 0.01, *** *P* < 0.001. Ctrl: healthy control samples; Case: PV samples.

Levels of total miRNAs were quantified the read count and TPM analyses. Compared with the control group, 246 differently expressed miRNAs (166 up-regulated and 80 down-regulated) were found in PV group (Figures 2A,B and Supplementary Table 3), including hsa-let-7d-3p, hsa-miR-125a-5p, hsa-miR-134-5p, hsa-miR-142-3p, hsa-miR-155-5p, hsa-miR-375-3p, hsa-miR-485-5p, hsa-miR-941, and hsa-miR-1228-5p. In addition, the heat map (Figure 3) revealed the differentially expressed miRNAs (*P* < 0.05) between the control group and the PV group. Besides, top differential miRNAs between PV patients and healthy control were stated in Table 2. Subsequently, qRT-PCR was performed to validate data of small RNA sequencing in 20 healthy control samples (control group) and 20 PV samples (case group). Except hsa-miR-125a-5p, hsa-miR-142-3p and hsa-miR-375-3p, qRT-PCR results of other miRNAs were consistent with those in small RNA sequencing (Figures 4A–I).

**TABLE 2:** Top differential miRNAs between psoriasis vulgaris (PV) patients and healthy control.

miRNA	TPM		Up/Down	Log2 (Fold change)
	HC	PV		
hsa-miR-222-5p	0.005	0.4845	Up	6.5984
hsa-miR-376b-3p	0.011	1.028	Up	6.5462
hsa-miR-449a	0.0028	0.174	Up	5.9575
hsa-miR-2115-5p	0.0171	0.8727	Up	5.6734
hsa-miR-4785	0.0055	0.2507	Up	5.5104
hsa-miR-4488	281.3985	4.1762	Down	–6.0743
hsa-miR-6513-3p	0.1356	0.004	Down	–5.0832
hsa-miR-4485-3p	1.3131	0.0482	Down	–4.7678
hsa-miR-4481	0.8671	0.0325	Down	–4.7377
hsa-miR-203a-3p	19.5013	0.8938	Down	–4.4475

*PV: psoriasis vulgaris; HC: healthy controls; TPM: transcripts per million.*

### Target Prediction of Differently Expressed miRNAs

**FIGURE 5:**
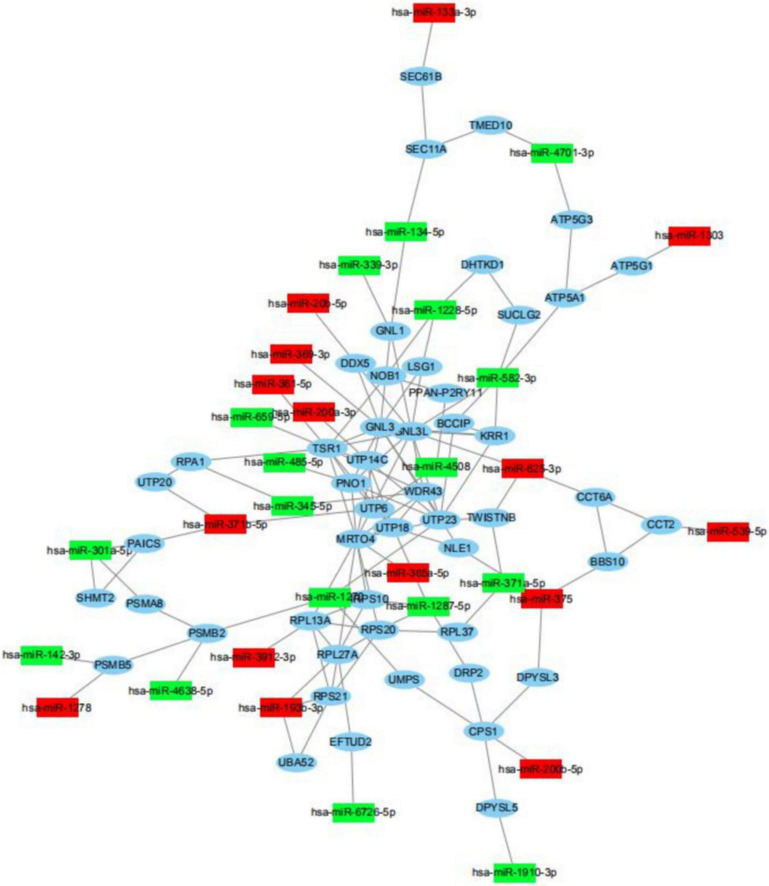
miRNA-mRNA regulatory network between differently expressed miRNAs and target genes. View of miRNA-mRNA regulatory network according to miRNAs with differently levels and their regulated target genes.

Usually, miRNAs play roles in biology progresses through regulating target gene expression. To understand the roles of differently expressed miRNAs responded to PV, target prediction was assessed. Target genes of differently expressed miRNAs were identified including DEAD-box helicase 5 (DDX5), SEC11 homolog A, signal peptidase complex subunit (SEC11A), TSR1 ribosome maturation factor (TSR1), ribosomal protein L13a (RPL13A), epidermal growth factor receptor (EGFR) and UTP6 small subunit processome component (UTP6) (Figure 5 and Supplementary Tables 4, 5). Moreover, miRNA-mRNA network indicated that a target gene could be modified both by up-regulated and down-regulated miRNAs (Figure 5).

### Functional Analysis of Differently Expressed miRNAs

**FIGURE 6:**
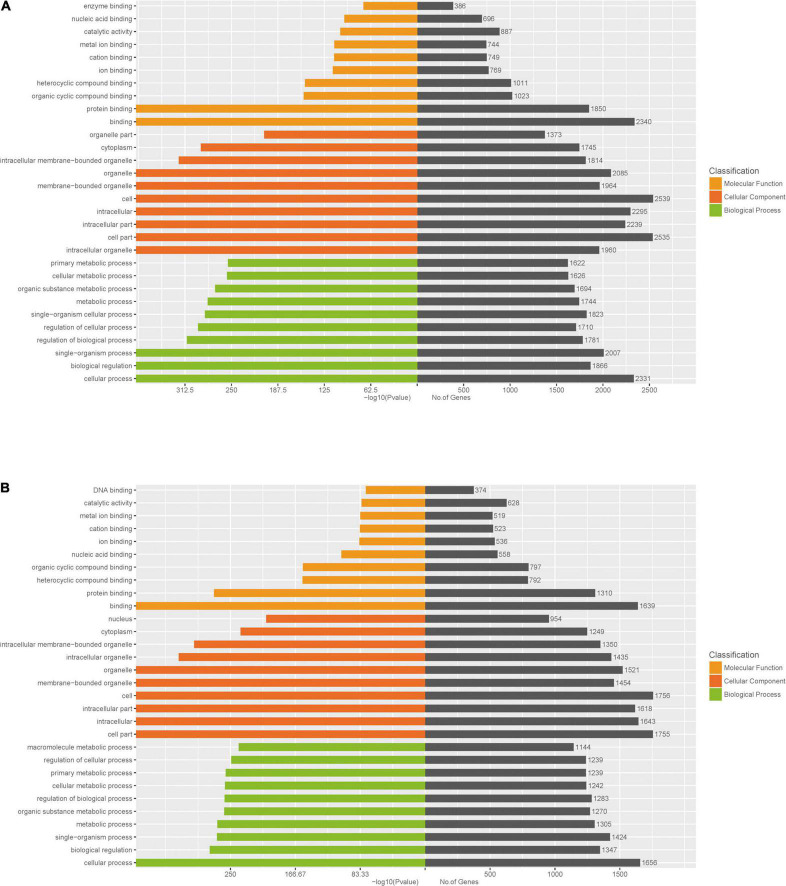
GO enrichment analysis for target genes of miRNAs with differently levels. The GO enrichment histograms and GO terms for target genes of up-regulated miRNAs **(A)** and down-regulated miRNAs **(B)** are shown.

**FIGURE 7:**
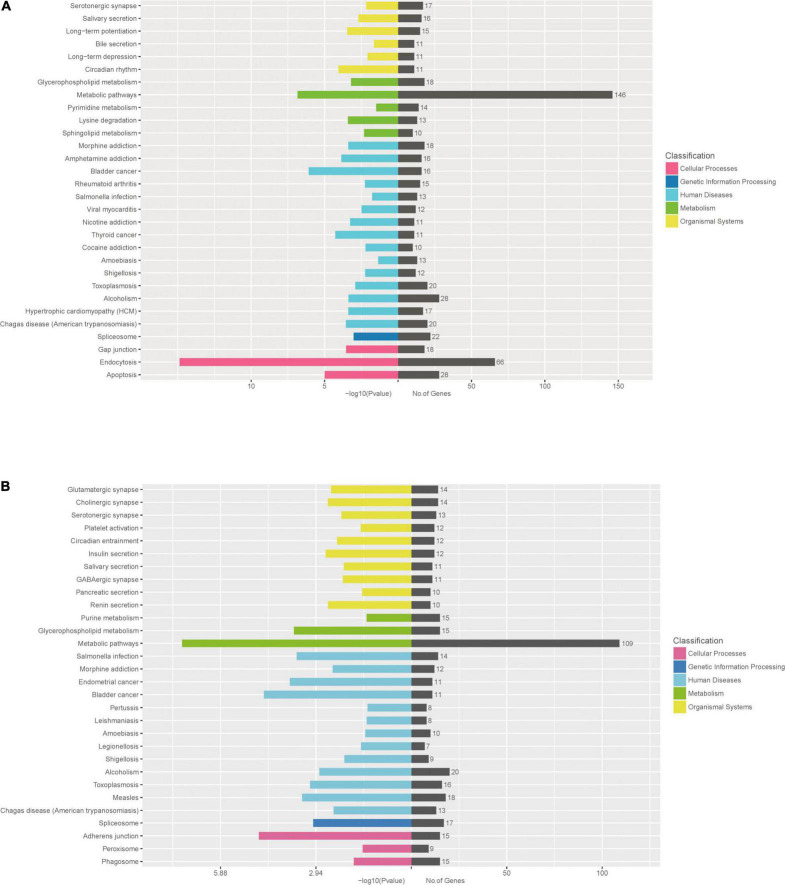
KEGG pathway enrichment analysis for target genes of miRNAs with differently levels. The KEGG pathway enrichment scatter plots for target genes of up-regulated miRNAs **(A)** and down-regulated miRNAs **(B)** are shown.

To further identify cellular processes and pathways related to differently expressed miRNAs, GO and KEGG pathway enrichment were further utilized to analyze their targets. GO enrichment analysis revealed that significantly enriched biological process for target genes of differently expressed miRNAs included primary metabolic process, cellular metabolic process, organic substance metabolic process, metabolic process, regulation of cellular process, signal-organism cellular process, regulation of biological process, biological regulation, cellular process (Figures 6A,B and Supplementary Table 6). In addition, the KEGG pathway enrichment analysis indicated that targets of up-regulated miRNAs were associated with metabolic pathways, endocytosis, apoptosis, alcoholism, spliceosome (Figure 7A and Supplementary Table 7), while targets of down-regulated miRNAs were involved in metabolic pathways, alcoholism, measies, spliceosome, toxoplasmosis (Figure 7B and Supplementary Table 7).

## Discussion

Here, we identified the levels of plasma exosomal miRNAs in PV patients. 1182 miRNAs including 336 novel miRNAs were investigated. In addition, 246 differently expressed miRNAs were identified including hsa-let-7d-3p, hsa-miR-125a-5p, hsa-miR-134-5p, hsa-miR-142-3p, hsa-miR-155-5p, hsa-miR-375-3p, hsa-miR-485-5p, hsa-miR-941and hsa-miR-1228-5p.

Previous studies have indicated that some of these differently expressed miRNAs associate with psoriasis. For example, serum hsa-miR-142-3p is significantly downregulated in patients with psoriasis after anti-tumor necrosis factor-α (TNF–α) therapy (24). In addition, hsa-miR-142-3p is highly upregulated in psoriatic skin (25). Moreover, miR-155 promotes proliferation and suppresses apoptosis of psoriasis cells (26), while treatment of methotrexate (MTX) and narrow-band ultraviolet B phototherapy (NB-UVB) decrease hsa-miR-155-5p expression in psoriatic skin lesions (27). Therefore, these miRNAs may exert influence on PV through exosome.

PV is an inflammatory skin disease (2, 28). Consistently, numerous of target genes of differently expressed miRNAs are involved in immunity. cAMP-response element binding protein 1 (CREB1), RUNX family transcription factor 2 (RUNX2) and epidermal growth factor receptor (EGFR) are targets of up-regulated miRNAs. CREB1 can activate the transcription of cytokine interleukin (IL) 33 as a transcription factor (29). CREB1 also enhances the production of IL-1 and TNF-α (30). Moreover, RUNX2 suppresses antitumor immunity in multiple myeloma cells (31). In contrast, RUNX2 contributes to clear viral infections through promoting IL-1 production in plasmacytoid dendritic cells (32). Recent studies have indicated that EGFR is a target of immunotherapy for tumors including lung cancer and glioblastoma multiforme (33–35).

Prediction of target genes revealed that insulin like growth factor binding protein 5 (IGFBP5), interleukin 13 receptor subunit alpha 1 (IL13RA1), cyclin D1 (CCND1) were modified by down-regulated miRNAs. IGFBP5 is essential for IL-6 production in human fibroblasts (36). IL-13Ralpha1 plays a critical role in immune responses for T helper type 2 -mediated disease (37). Furthermore, CCND1 is a target of immunotherapy for numerous cancers (38–40). Thus, plasma exosomal miRNAs may contribute to the inflammatory response in PV patients.

However, analysis of GO and KEGG pathway for target genes of differently expressed miRNAs demonstrated that these target genes were not enriched in inflammatory response or immunity but metabolic processes and metabolism pathways, indicating that most of target genes associated with metabolism. Metabolism fundamentally influences inflammatory response and ultimately affect progression of numerous diseases (41). T lymphocytes (T cells) are sentinels of immune system, and cellular metabolism activates T cell upon immune challenge through regulating blast, proliferation and differentiation (41). T cell metabolism is dynamically regulated with activation state (42). Upon antigen encounter, T cells are activated in a high rate of glycolysis for extensive proliferation and differentiation into effector. After pathogen clearance, most of effector T cells die while a few antigen-specific memory T cells were maintained (41–45). In addition, metabolism may contribute to the transition of effector T cells to memory T cells (46–48).

In detail, iron metabolism contributes to the proliferation of immune cells and cytokine action (49), consistent with the enriched GO terms “iron binding” and “metal iron binding.” Moreover, cholinergic metabolism is essential for vagus nerve-mediated immune function and proinflammatory responses (50). Furthermore, arginine metabolism, Vitamin D metabolism, Zinc metabolism, Myo-Inositol metabolism are crucial for immune cell growth and immunity (51–54). More importantly, some of immunity-related target genes in this study also contribute to metabolism. For example, CREB1 suppresses hepatic glucose metabolism (55). RUNX2 alters nutrient metabolism including glucose metabolism in cancers (56, 57). EGFR also regulates glucose metabolism in chondrosarcomas (58). Thus, plasma exosomal miRNAs may regulate immunity through modifying metabolism in PV patients.

However, the small size of the patient group was the limitation of this study. More PV patients would be recruited in the future study.

## Conclusion

In summary, the present study revealed candidate plasma exosomal miRNAs associated with PV and the signaling pathways modulated by miRNAs. These findings could provide potential biomarkers for diagnosis of PV and targets for clinical therapies against PV. However, the small size of the patient group was the limitation of this study. More PV patients would be recruited in the future study.

## Data Availability Statement

The original contributions presented in the study are included in the article/Supplementary Material, further inquiries can be directed to the corresponding authors.

## Ethics Statement

The studies involving human participants were reviewed and approved by Institutional Review Board of Guangdong Provincial Hospital of Chinese Medicine. The patients/participants provided their written informed consent to participate in this study.

## Author Contributions

X-MC, D-NY, R-YH, and C-JL: study conception and design and draft manuscript preparation. X-MC, D-NY, X-DW, and M-JW: data collection. X-MC, D-NY, X-DW, M-JW, J-WD, and HD: analysis and interpretation of results. All authors reviewed the results and approved the final version of the manuscript.

## Conflict of Interest

The authors declare that the research was conducted in the absence of any commercial or financial relationships that could be construed as a potential conflict of interest.

## Publisher’s Note

All claims expressed in this article are solely those of the authors and do not necessarily represent those of their affiliated organizations, or those of the publisher, the editors and the reviewers. Any product that may be evaluated in this article, or claim that may be made by its manufacturer, is not guaranteed or endorsed by the publisher.
