# Corrigendum: Deep sequencing of plasma exosomal microRNA level in psoriasis vulgaris patients

**DOI:** 10.3389/fmed.2023.1217484

**Published:** 2023-08-18

**Authors:** Xiu-Min Chen, Dan-Ni Yao, Mao-Jie Wang, Xiao-Dong Wu, Jing-Wen Deng, Hao Deng, Run-Yue Huang, Chuan-Jian Lu

**Affiliations:** ^1^State Key Laboratory of Dampness Syndrome of Chinese Medicine, The Second Affiliated Hospital of Guangzhou University of Chinese Medicine (Guangdong Provincial Hospital of Chinese Medicine), Guangzhou, China; ^2^The Second Affiliated Hospital, Guangzhou University of Chinese Medicine (Guangdong Provincial Hospital of Chinese Medicine), Guangzhou, China; ^3^Guangdong Provincial Key Laboratory of Chinese Medicine for Prevention and Treatment of Refractory Chronic Diseases, Guangzhou, China; ^4^Guangdong-Hong Kong-Macau Joint Lab on Chinese Medicine and Immune Disease Research, Guangzhou University of Chinese Medicine, Guangzhou, China

**Keywords:** psoriasis vulgaris, plasma, exosome, miRNA, inflammatory response, metabolism

In the published article, there was an error. It should be “plasma,” not “serum.” A correction has been made throughout the article: to the Article title, the Keywords, Abstract, Introduction, Materials and Methods, Discussion, and Conclusion, and are listed below.

In addition, there was an error where it should be **“**circulating” not “serum.” A correction has been made to the **Introduction** (paragraphs 2 and 3) and are listed below.

The **Article title** has been corrected to: “*Deep sequencing of plasma exosomal microRNA level in psoriasis vulgaris patients*.”

The **Keywords** have been corrected to: psoriasis vulgaris, plasma, exosome, miRNA, inflammatory response, metabolism.

A correction has been made to the **Abstract**. The sentence previously stated:

“Serum exosomal miRNAs have been identified as the reliable biomarkers and therapy targets of human diseases. Here, we described the levels of serum exosomal miRNAs in PV patients and analyzed the functional features of differently expressed miRNAs and their potential target genes for the first time. We identified 1182 miRNAs including 336 novel miRNAs and 246 differently expressed miRNAs in serum exosomes of healthy people and PV patients.”

The corrected sentence appears below:

“Plasma exosomal miRNAs have been identified as the reliable biomarkers and therapy targets of human diseases. Here, we described the levels of plasma exosomal miRNAs in PV patients and analyzed the functional features of differently expressed miRNAs and their potential target genes for the first time. We identified 1,182 miRNAs including 336 novel miRNAs and 246 differently expressed miRNAs in plasma exosomes of healthy people and PV patients.”

A correction has been made to **Introduction**, paragraphs 2 and 3. The sentences previously stated:

“Of these, serum exosomal miRNAs have been identified as the reliable biomarkers and therapy targets of human diseases, such as cancers, respiratory illness, diabetic nephropathy and autoimmune diseases (11–14).”

“Furthermore, serum exosomal miRNA miR-126 have potential to predict acute respiratory distress syndrome (16).”

“More importantly, a recent study has demonstrated that extrinsic microRNA *let-7i* transferred by serum exosomes might have an active role in triggering autoimmune diseases (18).”

“Considering that plasma exosomal microRNAs modulate immune response (11), serum exosomal microRNAs might have the potential to predict immune disorders including psoriasis vulgaris.”

“In this study, the high-throughput RNA sequencing was employed to identify differentially expressed serum exosomal miRNAs in patients with psoriasis vulgaris, and results were validated by quantitative real-time polymerase chain reaction (qRT-PCR).”

The corrected sentences appear below:

“Of these, circulating exosomal miRNAs have been identified as the reliable biomarkers and therapy targets of human diseases, such as cancers, respiratory illness, diabetic nephropathy and autoimmune diseases (11–14).”

“Furthermore, plasma exosomal miRNA miR-126 have potential to predict acute respiratory distress syndrome (16).”

“More importantly, a recent study has demonstrated that extrinsic microRNA *let-7i* transferred by plasma exosomes might have an active role in triggering autoimmune diseases (18).”

“Considering that circulating exosomal microRNAs modulate immune response (11), plasma exosomal microRNAs might have the potential to predict immune disorders including psoriasis vulgaris.”

“In this study, the high-throughput RNA sequencing was employed to identify differentially expressed plasma exosomal miRNAs in patients with psoriasis vulgaris, and results were validated by quantitative real-time polymerase chain reaction (qRT-PCR).”

A correction has been made to **Materials and Methods**, “*Exosome Isolation*.” The sentence previously stated:

“Exosomes were isolated from 500μl serum samples according to the manufacturer's protocols using Exo Quick Exosome Precipitation Solution Kit (20), and identified by scanning electron microscopy (SEM) (FEI XL30, The Netherlands) with low-voltage (1 KeV) and magnification of 20,000, NanoSight and Western blot analysis in our previous study (20), which shared exosomes used in the present study.”

The corrected sentence appears below:

“Exosomes were isolated from 500 μl plasma samples according to the manufacturer's protocols using Exo Quick Exosome Precipitation Solution Kit (20), and identified by scanning electron microscopy (SEM) (FEI XL30, The Netherlands) with low-voltage (1 KeV) and magnification of 20,000, NanoSight and Western blot analysis in our previous study (20), which shared exosomes used in the present study.”

A correction has been made to **Materials and Methods**, “*Small RNA Library Construction, Sequencing, and miRNA Identification*,” paragraph 1. The sentence previously stated:

“After the extraction of total RNA from serum exosome by TRIzol (Thermo Fisher Scientific, Waltham, MA, United States), the RNAs ranged from 18 to 30bp were enriched. Then adapters were ligated to RNAs followed by the reverse transcription of adapter-ligated RNAs, and the 140–160bp size products were collected for the construction of cDNA library and sequencing by Illumina HiSeq™ 4000.”

The corrected sentence appears below:

“After the extraction of total RNA from plasma exosome by TRIzol (Thermo Fisher Scientific, Waltham, MA, United States), the RNAs ranged from 18 to 30 bp were enriched. Then adapters were ligated to RNAs followed by the reverse transcription of adapter-ligated RNAs, and the 140–160 bp size products were collected for the construction of cDNA library and sequencing by Illumina HiSeq™ 4000.”

A correction has been made to **Discussion**, paragraphs 1, 4, and 6. These sentences previously stated:

“Here, we identified the levels of serum exosomal miRNAs in PV patients.”

“Thus, serum exosomal miRNAs may contribute to the inflammatory response in PV patients.”

“Thus, serum exosomal miRNAs may regulate immunity through modifying metabolism in PV patients.”

The corrected sentences appear below:

“Here, we identified the levels of plasma exosomal miRNAs in PV patients.”

“Thus, plasma exosomal miRNAs may contribute to the inflammatory response in PV patients.”

“Thus, plasma exosomal miRNAs may regulate immunity through modifying metabolism in PV patients.”

A correction has been made to **Conclusion**. The sentence previously stated:

“In summary, the present study revealed candidate serum exosomal miRNAs associated with PV and the signaling pathways modulated by miRNAs.”

The corrected sentence appears below:

“In summary, the present study revealed candidate plasma exosomal miRNAs associated with PV and the signaling pathways modulated by miRNAs.”

In the published article, there was an error in Figure 2 as published. The meaning of Figure 2A is the same as Figure 2B, which should be removed from Figure 2. The corrected Figure 2 and its caption appear below.

**Figure 2:**
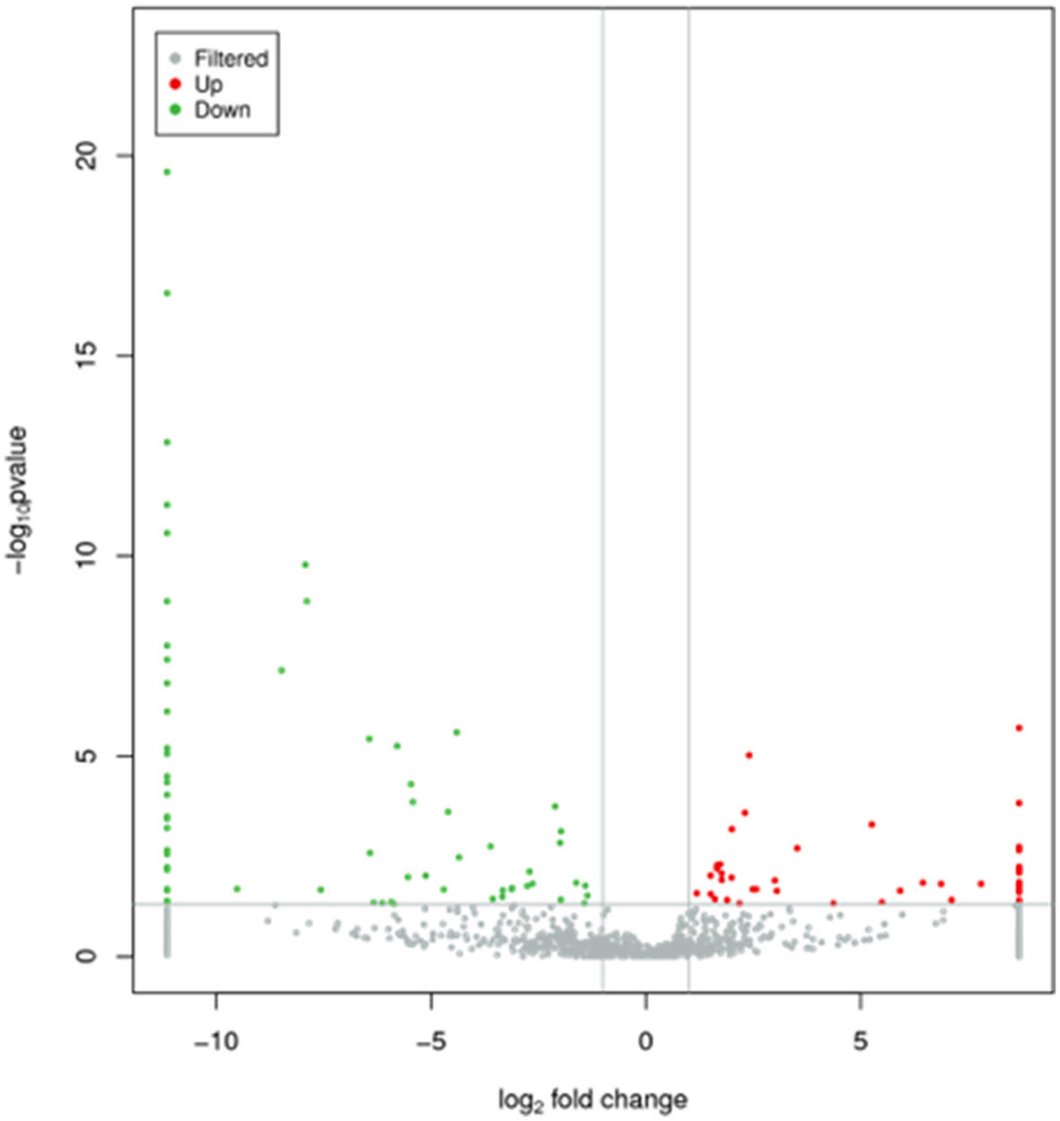
Characteristics of miRNA levels between control group and PV group. All miRNA levels are shown, and miRNAs with differentially levels are shown in red (up-regulated) or green (down-regulated).

In the published article, there was an error in Figure 3 as published. The groups in Figure 3 are a little confusing, which should be clustered again. The corrected Figure 3 and its caption appear below.

**Figure 3:**
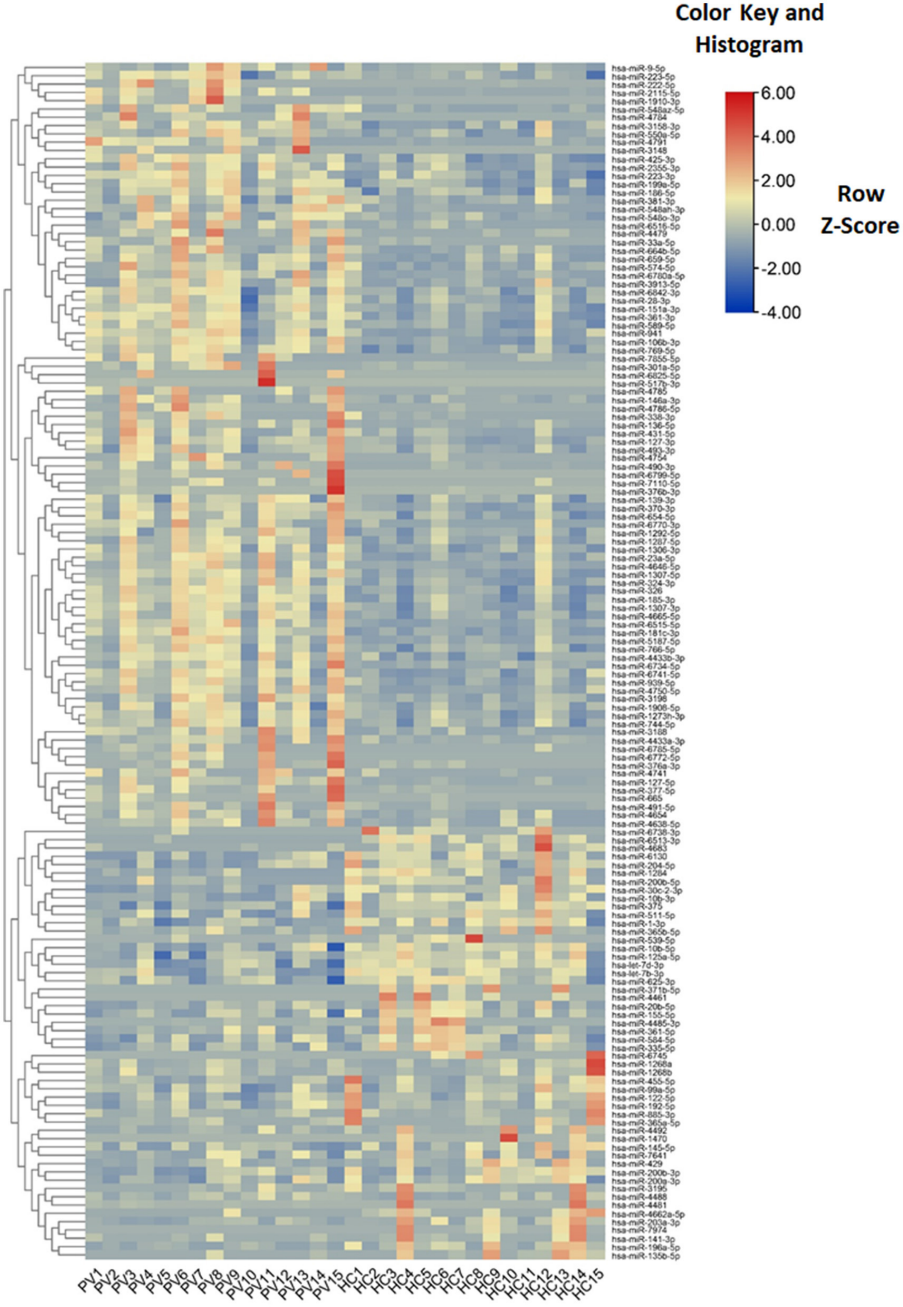
Characteristics of miRNA levels between different groups. Heat map showing the levels of miRNAs (*P* < 0.05) in different groups. Colors from blue to red stand for *z*-score got through the dimensionality reduction of FPKM value and reveal decreasing miRNA levels in each group.

The authors apologize for these errors and state that this does not change the scientific conclusions of the article in any way. The original article has been updated.

